# The usability of robotic-assisted systems for total knee arthroplasty can be improved without hindering the accuracy of the bone cuts

**DOI:** 10.1007/s00402-025-05818-8

**Published:** 2025-05-27

**Authors:** Killian Cosendey, Julien Stanovici, Jaad Mahlouly, Patrick Omoumi, Brigitte M. Jolles, Julien Favre

**Affiliations:** 1 Department of Orthopedic Surgery and Traumatology, Lausanne University Hospital and University of Lausanne (CHUV-UNIL), Lausanne, Switzerland; 2 Department of Diagnostic and Interventional Radiology, Lausanne University Hospital and University of Lausanne (CHUV-UNIL), Lausanne, Switzerland; 3 Institute of Electrical and Micro Engineering, Ecole Polytechnique Fédérale de Lausanne (EPFL), Lausanne, Switzerland; 4 The Sense Innovation and Research Center, Sion, Lausanne, Switzerland

**Keywords:** Total knee arthroplasty, Robotic-assisted surgery, Positional and angular errors, Registration

## Abstract

**Introduction:**

This study assessed the bone cuts accuracy of a robotic-assisted system for total knee arthroplasty (TKA) that was recently upgraded.

**Materials and methods:**

Three orthopaedic surgeons planned and executed TKA on 24 sawbones. Bone cut accuracy was assessed using CT scans, comparing the planning and the actual bone cuts in all six degrees-of-freedom.

**Results:**

The root-mean-square (RMS) values were below 2 mm or 2° for all error types, except for the medio-lateral position (2.4 mm) and internal-external rotation (2.3°) of the left tibias. The maximal amplitude of the 288 errors (6 degrees-of-freedom * 2 bones * 24 knees) was observed in tibial external rotation (3.2°). Most error types reported a bias, with limited variations among knees.

**Conclusions:**

The errors were in the same range as those of the prior version of the system, suggesting that the improvements brought by the system upgrade were not obtained at the expense of accuracy.

## Introduction

Robotic assistance is increasing in Total Knee Arthroplasty (TKA), particularly because it offers the possibility to improve the accuracy of the bone cuts compared to conventional methods [1–4]. This field is highly dynamic, with regular updates of the robotic tools. To confirm the improvement over time, it is therefore important to keep evaluating the new releases.

**Fig. 1:**
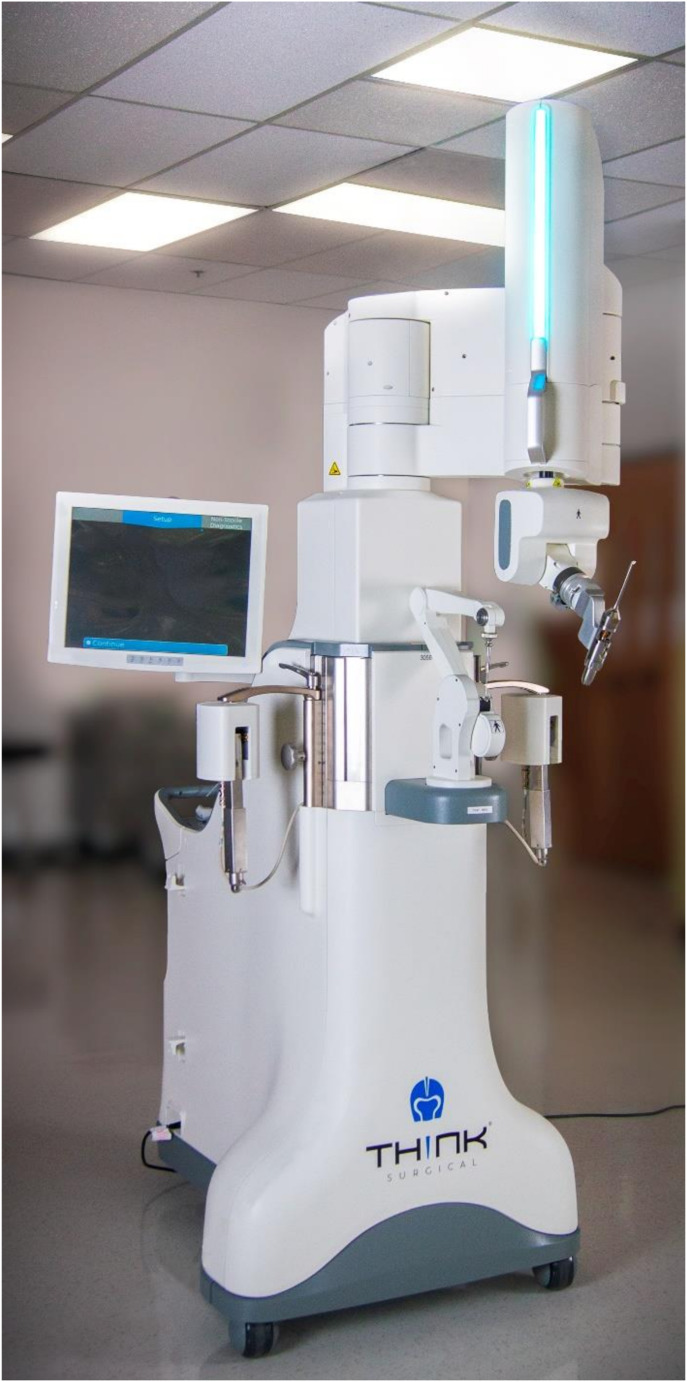
Robotic-assisted surgery device (310 TCAT®, TSOLUTION ONE® Total Knee Application, Think Surgical Inc., Fremont, CA, USA)

Recently, THINK Surgical Inc. (California, USA) released a new version of its *TSOLUTION ONE*^*®*^ system (*310* vs. *300* previously, Fig. 1), where both the robotic-assisted surgery device that cuts the bone autonomously (*310 TCAT*^*®*^) and the planning workstation (*310 TPLAN*^*®*^) were upgraded. These upgrades bring a series of improvements, including faster calibration and registration procedures, as well as extended planning possibilities allowing a complete control of the implant placements in all six degrees of freedom. Although appreciable, it is necessary to ensure that these improvements did not lower the accuracy of the cuts that was shown to be appropriate with the 300 version [5]. Furthermore, when assessing the newer version of the system, it would be interesting to test both left and right knees and determine whether the accuracy varies by side. In fact, whereas various aspects could lead to accuracy differences between left and right knees, there is a paucity of data in this regard in literature [6, 7].

The purpose of this study was to assess the bone cuts accuracy of the 310 TSOLUTION ONE system in terms of position and orientation. The study also aimed to determine if the accuracy differs between left and right knees.

## Materials and methods

This study used the same methodology and involved the same three orthopaedic surgeons as an earlier work assessing the 300 version [5]. The orthopaedic surgeons were trained on the newer version of the system and each surgeon performed TKA on four right and four left sawbone knees (medium size, solid foam material, Pacific Research Company, Vashon Island, Washington), following the manufacturer’s recommendations. A sample size calculation based on the accuracy of the 300 version [5] indicated that groups of 12 knees were sufficient to detect left-right difference of at least 1 mm or 1° with a significance level of 5% and a power of 80%. The detection threshold was selected conservatively as smaller differences are certainly clinically irrelevant [8].

**Fig. 2:**
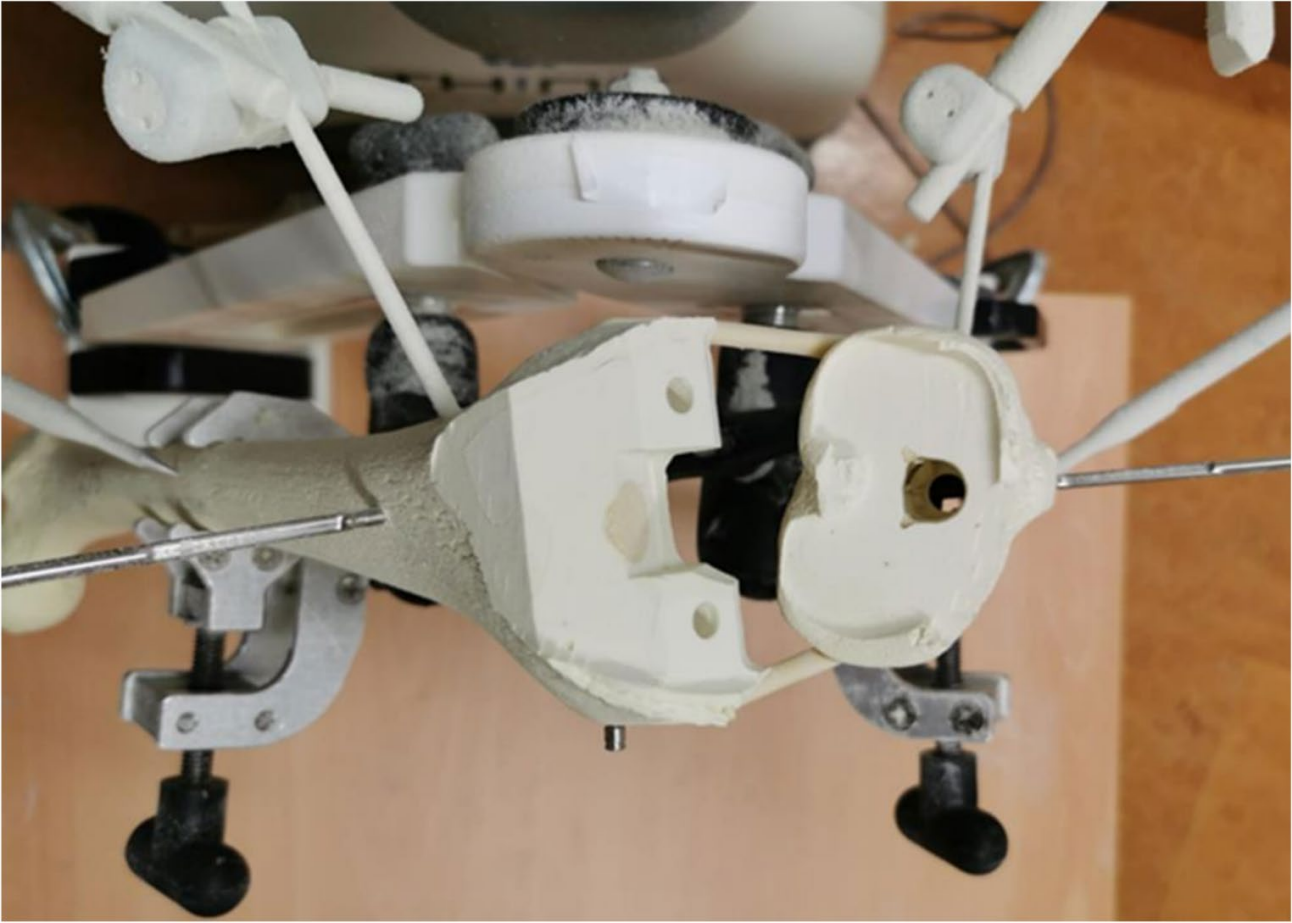
Example of a cut knee

To assess the accuracy, 10 fiducial markers (metallic beads of 0.8 mm diameter) were embedded in each femur and tibia sawbones. The sawbones were then CT scanned using a Discovery CT750 HD machine (GE Healthcare, Chicago, USA) parametrized as follows: field of view of 250 × 250 mm, matrix size of 512 × 512 pixels, slice thickness of 0.312 mm, tube voltage of 120 kVp and tube current of 200 mAs. The resulting images were uploaded to the planning workstation to reconstruct the 3D bone models, enabling the surgeons to plan the TKA. Then, the sawbones were cut autonomously by the robotic-assisted device (Fig. 2). After the cuts, the sawbones were CT scanned a second time using the same parameters as the initial scan, except for the slice thickness (0.625 mm) and the 3D models of the cut sawbones were reconstructed using in-house software [9]. Then, the model of the original and cut sawbones were imported into Matlab (Mathworks, Natick, MA, USA) and registered using the fiducial markers. Separately, for both the tibia and femur, the registration was performed by identifying the center of the markers and computing the transformation that aligned the markers from the cut sawbone to those on the original sawbone [10]. To quantify the accuracy, reference frames were defined for the femoral and tibial cuts, based on the geometry of the cuts. Using the registered data, these reference frames were aligned to both the planned cuts on the original sawbones and the actual cuts. Finally, for each bone, the differences between the planned and actual reference frames were calculated for the femur and the tibia. The positional accuracy of the cut relative to the planning was measured along the medial-lateral, anterior-posterior and proximal-distal axes. Similarly, the angular accuracy was measured in the sagittal, frontal and transverse planes. All procedures were planned with “Persona Posterior Stabilized” components (Zimmer, Warsaw, Indiana).

### Statistical analysis

Results were reported using median and interquartile range (IQR). The root-mean-square (RMS) errors and the percentage of outlier cuts, meaning cuts with errors exceeding +/- 3 mm or +/- 3° [11], were also reported in agreement with the literature. Wilcoxon rank sum tests were conducted to compare errors in left and right knees, whereas Wilcoxon signed-rank tests were performed to detect errors different from zero, therefore indicating biases in the cut. Comparisons with errors in an earlier study assessing the 300 version of the system on 30 right knees [5] was also performed using Wilcoxon rank sum tests. The significance level was set a priori to 5%.

The reliability of the error measurement method was already assessed in a previous study [5]. Two randomly selected knees were CT-scanned and processed five times each by the same operator. The results demonstrated RMS differences among repeats under 0.2 mm and 0.3° for all positional and angular errors.

## Results

**Fig. 3:**
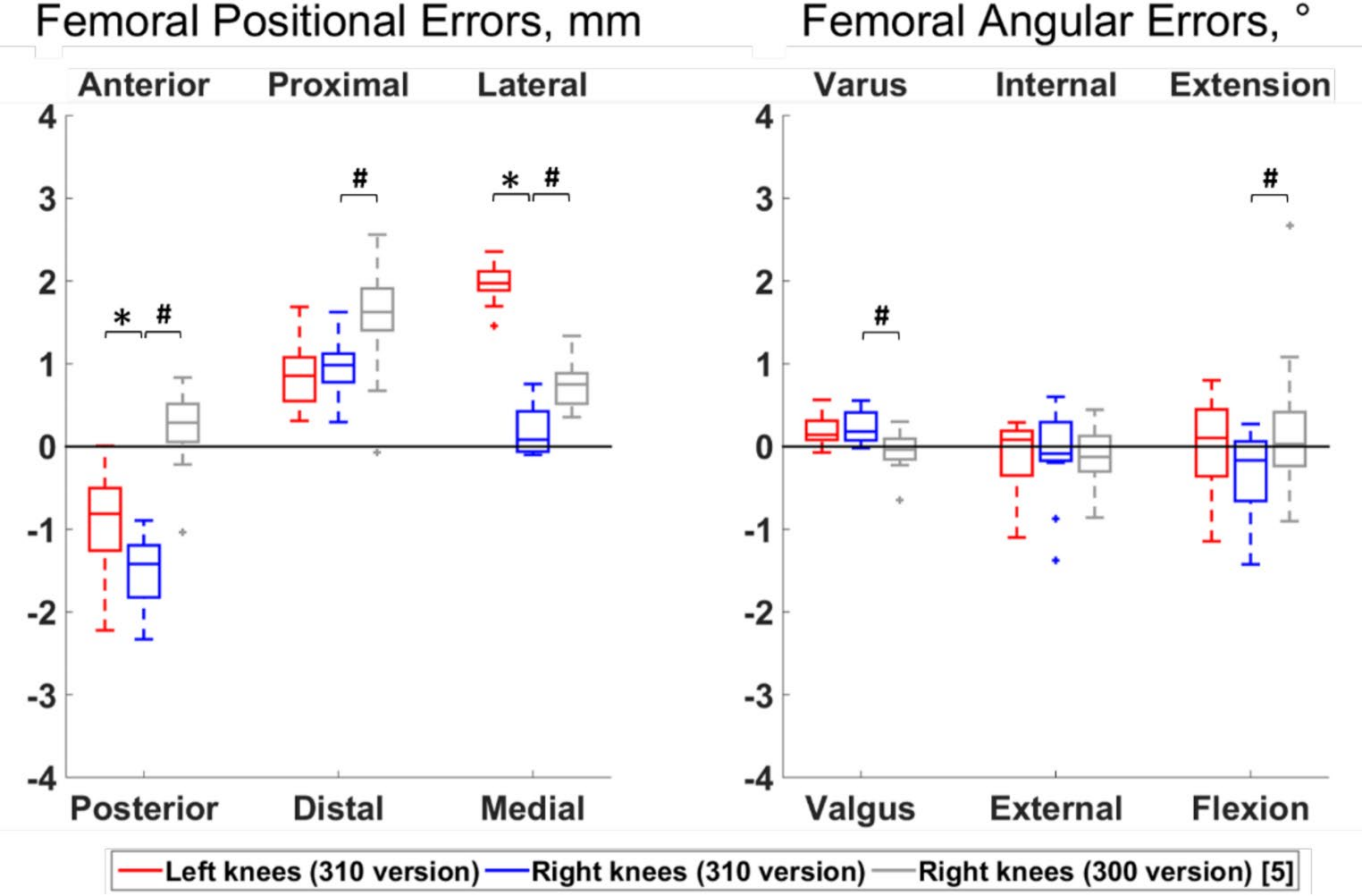
Boxplot of the positional and angular errors for the femoral cut. Results from the present study on the newer version of the system (310 TSOLUTION ONE) are plotted in red for the left knees (n = 12) and in blue for the right knees (n = 12). Statistically significant differences among sides are indicted by stars (*p* < 0.05). For completeness, the errors from a prior work [5] assessing the earlier version of the system (300 TSOLUTION ONE) on right knees (n = 30) are displayed in grey. Statistically significant differences in right knee errors between both versions of the systems are indicated by hashes (*p* < 0.05). The directions at the top and bottom of the plots indicate where the actual cuts were comparted to the planned cuts. For example, a positive anterior-posterior error indicates a cut that was too anterior compared to the planning

**Fig. 4:**
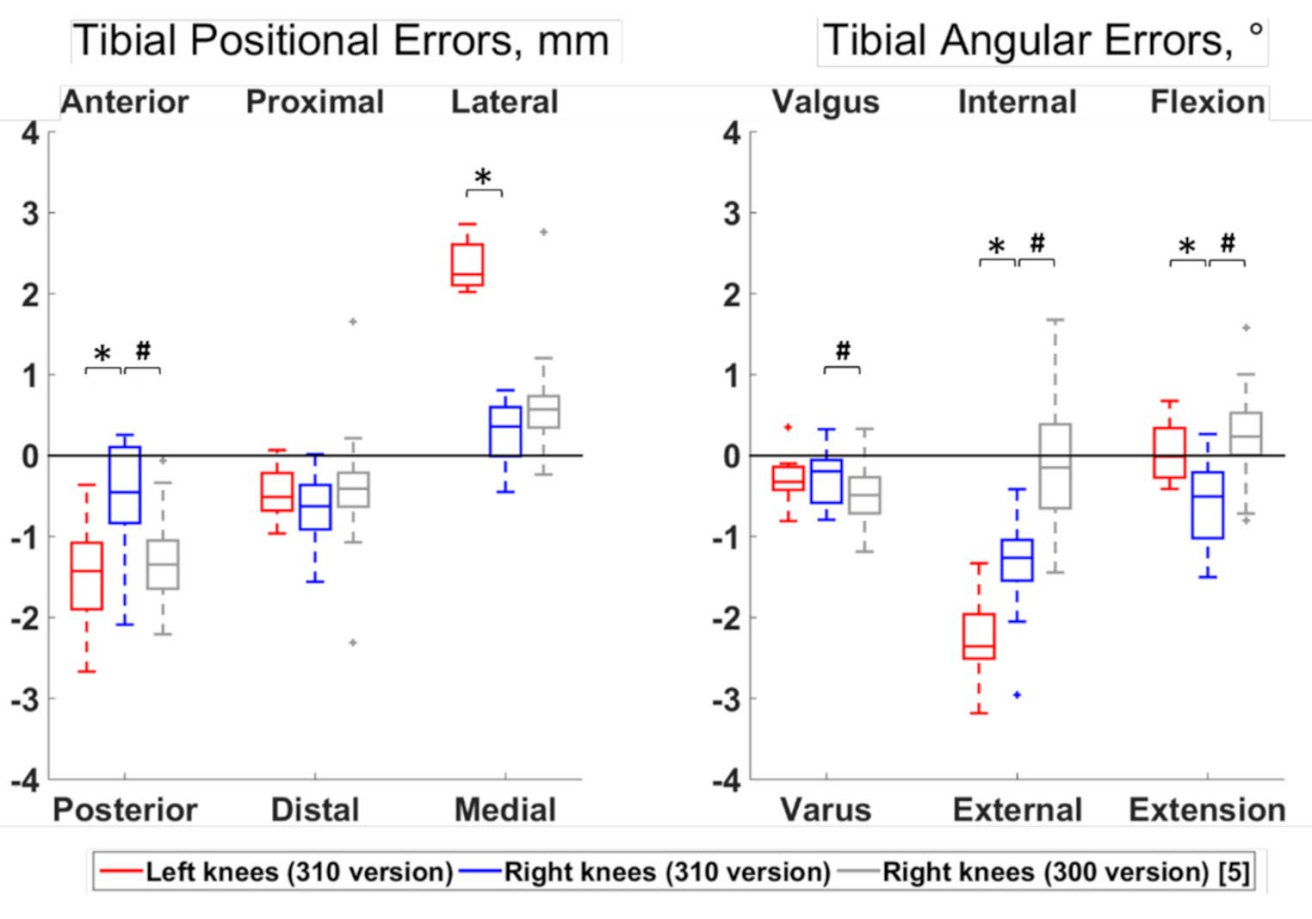
Boxplot of the positional and angular errors for the tibial cut. Results from the present study on the newer version of the system (310 TSOLUTION ONE) are plotted in red for the left knees (n = 12) and in blue for the right knees (n = 12). Statistically significant differences among sides are indicted by stars (*p* < 0.05). For completeness, the errors from a prior work [5] assessing the earlier version of the system (300 TSOLUTION ONE) on right knees (n = 30) are displayed in grey. Statistically significant differences in right knee errors between both versions of the systems are indicated by hashes (*p* < 0.05). The directions at the top and bottom of the plots indicate where the actual cuts were comparted to the planned cuts. For example, a positive anterior-posterior error indicates a cut that was too anterior compared to the planning

Differences between left and right knees were observed for 6 out of the 12 error types (Figs. 3 and 4). Specifically, the right femoral cuts were more posterior and medial compared to the left cuts (median differences of 0.61 mm and 1.89 mm, respectively; *p* ≤ 0.05), and the right tibial cuts were more anterior, medial, internally rotated and extended compared to the left cuts (median differences of 0.97 mm, 1.88 mm, 1.09° and 0.49°, respectively; *p* ≤ 0.05).

The RMS values of all error types were below 2 mm or 2° (Table 1), except for the medio-lateral (2.36 mm) and internal-external (2.34°) errors of the left tibias. In addition, 2 out of the 24 knees had one error qualifying as outliers, both towards tibial external rotation (3.03° and 3.18°).

**Table 1:** Positional and angular errors of left (*n* = 12) and right (*n* = 12) knees

Error type	Side ^#^	Median {IQR}	RMS	Percentage of outliers
Femur				
Positional errors, mm				
Anterior (+) / posterior (-) ^γ^	Left knees	-0.81 {0.54}*	1.09	0
	Right knees	-1.42 {0.51}*	1.59	0
Proximal (+) / distal (-)	All knees	0.90 {0.37}*	0.99	0
Lateral (+) / medial (-) ^γ^	Left knees	1.97 {0.22}*	1.99	0
	Right knees	0.08 {0.46}	0.32	0
Angular errors, °				
Varus (+) / Valgus (-)	All knees	0.17 {0.27}*	0.28	0
Internal (+) / External (-)	All knees	0.02 {0.43}	0.51	0
Extension (+) / Flexion (-)	All knees	-0.13 {0.75}	0.61	0
Tibia				
Positional errors, mm				
Anterior (+) / posterior (-) ^γ^	Left knees	-1.43 {0.77}*	1.6	0
	Right knees	-0.45 {0.90}	0.85	0
Proximal (+) / distal (-)	All knees	-0.51 {0.49}*	0.69	0
Lateral (+) / medial (-) ^γ^	Left knees	2.24 {0.46}*	2.36	0
	Right knees	0.36 {0.53}*	0.51	0
Angular errors, °				
Valgus (+) / Varus (-)	All knees	-0.28 {0.37}*	0.40	0
Internal (+) / External (-) ^γ^	Left knees	-2.36 {0.51}*	2.34	17
	Right knees	-1.26 {0.46}*	1.51	0
Flexion (+) / Extension (-) ^γ^	Left knees	-0.01 {0.49}	0.37	0
	Right knees	-0.51 {0.70}*	0.78	0

Median, interquartile range (IQR) and root-mean-square (RMS) data are in mm for positional errors and in degree for angular errors

^#^: In the absence of statistically significant difference between sides, errors were calculated for left and right knees together

γ: Statistically significantly different between sides (*p* ≤ 0.05)

*: Statistically significantly different from zero (*p* ≤ 0.05)

The values in this table indicate where the actual cuts were comparted to the planned cuts

Apart from the flexion-extension and internal-external rotation of the femur, biases were observed with all error types (*p* ≤ 0.05). The median value of the error types reporting a bias were below 1 mm or 1°, except for the femoral cuts that were too posterior by 1.42 mm in right knees and too lateral by 1.97 mm in left knees and for the tibial cuts that were too posterior by 1.43 mm, lateral by 2.24 mm and externally rotated by 2.36° in left knees and too externally rotated by 1.26° in right knees.

Statistically significant differences between right knees processed with the 300 and 310 versions of the system were observed for 9 out of the 12 error types (Figs. 2 and 3). Specifically, the errors were of smaller amplitudes with the 310 version for the femoral proximal-distal and medio-lateral positions and for the tibial antero-posterior position and varus-valgus (median differences of 0.64 mm, 0.67 mm, 0.89 mm and 0.29°, respectively; *p* ≤ 0.05). In contrast, the errors had smaller amplitudes with the 300 version for the femoral antero-posterior position, varus-valgus and flexion-extension, as well as for the tibial internal-external rotation and flexion-extension (median differences of 1.71 mm, 0.22°, 0.19°, 1.12° and 0.74°, respectively; *p* ≤ 0.05).

## Discussion

With only 2 out of the 288 errors (24 knees x 12 types of error) classified as outliers and RMS values below 1 mm or 1° for half the error types, the 310 TSOLUTION ONE system showed its capacity to achieve accurate bone cuts. Furthermore, biases were observed, especially in the error types showing the largest RMS values, suggesting that the accuracy could be improved by identifying and annulling the source of systematic deviations. The errors differed between left and right knees and looking more closely at these findings indicated that the side differences were in fact a consequence of different biases in left and right knees. Therefore, reducing the biases would certainly lower the amplitudes and the side differences of the errors at the same time. Further work would be necessary to determine the source of biases and manage them, possibly acting on the calibration or registration procedures. Nevertheless, the biases were relatively small and the impact of such errors on the clinical outcomes remain unknown [12]. Moreover, this study tested a single system once. Therefore, before engaging in any system modification, the variations in errors among systems and over time should probably be characterized. Until this has been done, it is important to consider the errors as indicative of accuracy ranges rather than as definitive values. Consequently, as presented above, a robust manner to summarize the accuracy of the system tested in this study is to report that the bone cut errors rarely exceed 3 mm or 3°, and are often below 2 mm or 2° or even below 1 mm or 1°.

Comparing the errors for the right knees in this study obtained with the 310 version to those from an earlier work on the 300 version [5] indicated globally similar results. Differences were observed, but they were too small and heterogeneous to suggest any compelling difference in the accuracy of the cuts achieved by both versions of the system. Nevertheless, looking at the results of both studies together reinforced the observations that the cuts are consistent from one knee to the other and that the errors are mostly biases.

Compiling data from bone cut accuracy studies of other robotic TKA systems provides valuable context, despite limitations due to methodological differences in robotic systems, sample sizes, knee conditions, and measurement techniques [13–17]. Generally, these systems have shown similar levels of accuracy, with prior studies reporting mean or median femoral bone cut accuracy between 0.1° and 1.1°, consistent with the 0.0° to 0.2° found in this study, and mean or median tibial bone cut accuracy between 0.1° and 0.7°, comparable to the 0.0° to 0.5° observed in the present study, except for tibial internal-external rotation (1.3° for right knees and 2.4° for left knees). This latter parameter is notably underreported in many studies [14–17], indicating a need for further research on this specific metric. Overall, these results highlight the effectiveness of robotic systems in achieving accurate bone cuts for TKA.

The use of sawbones was advantageous for the registration of the cut bones on the preoperative planning as fiducial markers could be embedded in the sawbones. Using sawbones also removed the natural variability among individual human knees, therefore resulting in standardized data that will facilitate systems comparison in the future. However, it is important to acknowledge the limitations associated with the use of sawbones. Unlike real pathological bones, which often present osteophytes, deformities, and varying stiffness, sawbones represent healthy bone structures and lack cortical, cancellous, or sclerotic bone characteristics. This discrepancy raises questions about the model’s ability to accurately reflect patient-specific conditions, potentially limiting the generalizability of the results to clinical settings. Nevertheless, it is important to note that the use of a robotic system in these procedures may mitigate some of the challenges posed by bone deformities. The precision and repeatability of robotic-assisted system could reduce the influence of anatomical irregularities, such as osteophytes or deformities, on the accuracy of the cuts. While these deformities may complicate manual procedures, the robotic system may perform consistently, regardless of such variations.

Given these considerations, future studies should incorporate more clinically representative models, including pathological bones with deformities, to better assess the system’s performance under real surgical conditions. Moreover, considering the preponderant role of biases in the errors and their variations among experimental conditions, further studies are encouraged to diversify the testing conditions, for example by assessing different copies of the system or different implants, in order to improve our understanding of the biases. Additional studies will also be necessary to determine the clinical implication of the biases.

## Conclusion

The bone cut errors of the new version of the TSOLUTION ONE system were in the same range as those of its prior version, suggesting that the improvements brought by the 310 version were not obtained at the expense of accuracy. This study also highlighted an important role of biases in the errors. On one hand, this suggested that the accuracy could be improved by reducing the systematic components of the errors. While encouraging, further research is necessary to evaluate the clinical impact of the bone cut errors and therefore the attention that should be paid to them in the future upgrades of the system. On the other hand, the different biases among experimental conditions recommend considering the errors as indicative of accuracy ranges rather than as definitive values.

## Data Availability

N.A.

## References

[1] Bautista M, Manrique J, Hozack WJ (2019) Robotics in total knee arthroplasty. J Knee Surg 32:600–606. 10.1055/s-0039-1681053 30822790 10.1055/s-0039-1681053

[2] Kayani B, Konan S, Ayuob A, Onochie E, Al-Jabri T, Haddad FS (2019) Robotic technology in total knee arthroplasty: A systematic review. EFORT Open Rev 4:611–617. 10.1302/2058-5241.4.190022 31754467 10.1302/2058-5241.4.190022 PMC6836078

[3] Khlopas A, Sodhi N, Sultan AA, Chughtai M, Molloy RM, Mont MA (2018) Robotic arm–assisted total knee arthroplasty. J Arthroplasty 33:2002–2006 29506926 10.1016/j.arth.2018.01.060

[4] Mart JPS, Goh EL (2021) The current state of robotics in total knee arthroplasty. EFORT Open Rev 6:270–279. 10.1302/2058-5241.6.200052 34040804 10.1302/2058-5241.6.200052 PMC8142057

[5] Cosendey K, Stanovici J, Mahlouly J, Omoumi P, Jolles BM, Favre J (2021) Bone cuts accuracy of a system for total knee arthroplasty including an active robotic arm. J Clin Med 10:3714. 10.3390/JCM10163714 34442008 10.3390/jcm10163714 PMC8397104

[6] Huang S, Li X, Tang Y, Stiphan S, Yan B, He P et al (2017) Different patient satisfaction levels between the first and second knee in the early stage after simultaneous bilateral total knee arthroplasty (TKA): A comparison between subjective and objective outcome assessments. J Orthop Surg Res 12:1–6. 10.1186/S13018-017-0605-0 28747231 10.1186/s13018-017-0605-0 PMC5530562

[7] Ishii Y, Noguchi H, Sato J, Takahashi I, Ishii H, Ishii R et al (2022) Comparison of operative times in primary bilateral total knee arthroplasty performed by a single surgeon. J Clin Med 11:4867. 10.3390/jcm11164867 36013109 10.3390/jcm11164867 PMC9410018

[8] Antoniadis A, Camenzind RS, Schär MO, Bergadano D, Helmy N (2019) Accuracy of tibial cuts with patient-specific instrumentation is not influenced by the Surgeon’s level of experience. Knee surgery. Sport Traumatol Arthrosc 27:1535–1543. 10.1007/s00167-018-4992-5 10.1007/s00167-018-4992-5 29872869

[9] Omoumi P, Babel H, Jolles BM, Favre J (2017) Quantitative regional and sub-regional analysis of femoral and tibial subchondral bone mineral density (sBMD) using computed tomography (CT): comparison of non-osteoarthritic (OA) and severe OA knees. Osteoarthr Cartil 25(11):1850–1857 10.1016/j.joca.2017.07.014 28743608

[10] Veldpaus FE, Woltring HJ, Dortmans LJMG (1988) A least-squares algorithm for the equiform transformation from Spatial marker co-ordinates. J Biomech 21(1):45–54 3339026 10.1016/0021-9290(88)90190-x

[11] Sikorski JM (2008) Alignment in total knee replacement. J Bone Joint Surg Br 90:1121–1127 18757949 10.1302/0301-620X.90B9.20793

[12] Parratte S, Pagnano MW, Trousdale RT, Berry DJ (2010) Effect of postoperative mechanical axis alignment on the fifteen-year survival of modern, cemented total knee replacements. J Bone Joint Surg Am 92:2143–2149. 10.2106/JBJS.I.01398 20844155 10.2106/JBJS.I.01398

[13] Sires JD, Craik JD, Wilson CJ (2021) Accuracy of bone resection in MAKO total knee robotic-assisted surgery. J Knee Surg 34(07):745–748 31694057 10.1055/s-0039-1700570

[14] Zaidi F, Goplen CM, Bolam SM, Monk AP (2024) Accuracy and outcomes of a novel Cut-Block positioning Robotic-Arm assisted system for total knee arthroplasty: A systematic review and Meta-Analysis. Arthroplasty Today 29:101451 39188576 10.1016/j.artd.2024.101451 PMC11345934

[15] Hampp EL, Chughtai M, Scholl LY, Sodhi N, Bhowmik-Stoker M, Jacofsky DJ, Mont MA (2019) Robotic-arm assisted total knee arthroplasty demonstrated greater accuracy and precision to plan compared with manual techniques. J Knee Surg 32(03):239–250 29715696 10.1055/s-0038-1641729

[16] Miao H, Zhu Z, Wang H, Bai X, Li X (2024) Predictive accuracy analysis of a novel robotic-assisted system for total knee arthroplasty: A prospective observational study. Ther Clin Risk Manag 473–482 10.2147/TCRM.S468598 PMC11318605 39135983

[17] Hasegawa M, Tone S, Naito Y, Sudo A (2024) Comparison of accuracy and early outcomes in robotic total knee arthroplasty using NAVIO and ROSA. Sci Rep 14(1):3192 38326363 10.1038/s41598-024-53789-4 PMC10850152

